# Coordinated Respiratory Motor Activity in Nerves Innervating the Upper Airway Muscles in Rats

**DOI:** 10.1371/journal.pone.0166436

**Published:** 2016-11-10

**Authors:** Satoshi Tachikawa, Kiyomi Nakayama, Shiro Nakamura, Ayako Mochizuki, Takehiko Iijima, Tomio Inoue

**Affiliations:** 1 Department of Oral Physiology, Showa University School of Dentistry, Shinagawa-ku, Tokyo 142–8555, Japan; 2 Department of Perioperative Medicine, Division of Anesthesiology, Showa University School of Dentistry, Oota-ku, Tokyo 145–8515, Japan; Texas Christian University, UNITED STATES

## Abstract

Maintaining the patency of the upper airway during breathing is of vital importance. The activity of various muscles is related to the patency of the upper airway. In the present study, we examined the respiratory motor activity in the efferent nerves innervating the upper airway muscles to determine the movements of the upper airway during respiration under normocapnic conditions (pH = 7.4) and in hypercapnic acidosis (pH = 7.2). Experiments were performed on arterially perfused decerebrate rats aged between postnatal days 21–35. We recorded the efferent nerve activity in a branch of the cervical spinal nerve innervating the infrahyoid muscles (CN), the hypoglossal nerve (HGN), the external branch of the superior laryngeal nerve (SLN), and the recurrent laryngeal nerve (RLN) with the phrenic nerve (PN). Inspiratory nerve discharges were observed in all these nerves under normocapnic conditions. The onset of inspiratory discharges in the CN and HGN was slightly prior to those in the SLN and RLN. When the CO_2_ concentration in the perfusate was increased from 5% to 8% to prepare for hypercapnic acidosis, the peak amplitudes of the inspiratory discharges in all the recorded nerves were increased. Moreover, hypercapnic acidosis induced pre-inspiratory discharges in the CN, HGN, SLN, and RLN. The onset of pre-inspiratory discharges in the CN, HGN, and SLN was prior to that of discharges in the RLN. These results suggest that the securing of the airway that occurs a certain time before dilation of the glottis may facilitate ventilation and improve hypercapnic acidosis.

## Introduction

Coordination of the patency of the upper airway with contraction of the diaphragm and intercostal muscles is important during breathing. The activity of various muscles is related to the patency of the upper airway, including the muscles of the tongue and lingual radix (such as the genioglossus, geniohyoid, and hyoglossus) innervated by the hypoglossal nerve (HGN), the infrahyoid muscles (sternothyroid, sternohyoid, thyrohyoid, and omohyoid) innervated by the ansa cervicalis receiving fibers from the HGN or 1st–3rd cervical spinal nerves (CN) [1, 2], the cricothyroid and inferior pharyngeal constrictor muscles innervated by the external branch of the superior laryngeal nerve (SLN) [3], and the laryngeal abductors (posterior cricoarytenoid muscle) and laryngeal adductors (lateral cricoarytenoid muscle and thyroarytenoid muscle) innervated by the recurrent laryngeal nerve (RLN) [3]. The respiratory motor activity in parts of these muscles and nerves has been previously examined in *in vivo* preparations. HGN activity is exhibited in the inspiratory phase and the onset of HGN activity occurs prior to the onset of phrenic nerve (PN) activity during eupnea [4, 5]. Furthermore, the activity of the posterior cricoarytenoid muscle and innervating RLN branch is also observed in the inspiratory phase; however, the activity of the thyroarytenoid muscle and innervating branch of the RLN are observed in the early expiratory phase [5, 6, 7]. Nonetheless, few studies have investigated the respiratory motor activity in the other muscles of the upper airway or in the nerves innervating those muscles despite the fact that coordinated and appropriate movement of the upper airway muscles is important in regulating the airflow through the upper airway during respiration.

Hypercapnic acidosis is a pathological condition induced by chronically obstructive pulmonary disease, such as bronchial asthma and sleep-related breathing disorders. Pharyngeal airway obstruction during sleep is proposed to be caused by a reduction in the inspiratory activity in the genioglossus [8]. When hypercapnic acidosis occurs, the augmentation of the expiratory drive is known to promote the excretion of CO_2_ [9, 10], and the activity pattern of the upper airway muscles is most likely to consequently change under such forced expiration.

Anesthesia is widely acknowledged to depress the respiratory rhythm and respiratory neural discharges. Respiratory electrical activity in the cranial nerves and their innervation of the upper airway muscles has been found to be more sensitive to anesthesia than activity in the phrenic nerve [11, 12]. Furthermore, some studies have reported that anesthesia also influences central chemosensitivity [13, 14]. Massey et al. [14] demonstrated that isoflurane reduces CO_2_ chemosensitivity due to the inhibition of the spontaneous firing of serotonin neurons, which are known to be CO_2_-sensitive. Thus, in this study, we examined the respiratory motor activity in the efferent nerves innervating the upper airway muscles using *in situ* arterially perfused decerebrate rat preparations [15, 16], enabling us to study the respiratory network without the depressant effects of anesthesia. This experimental model has been used in a multitude of studies to examine the respiratory network because the respiratory rhythm [15] and firing patterns of the medullary respiratory neurons [17] are similar to those of adult *in vivo* mammal preparations. We also analyzed the differences in the burst onset of each nerve during eupnea and forced respiration to verify the hypothesis that the activity pattern of the upper airway muscles changes under forced expiration to promote the excretion of CO_2_.

## Materials and Methods

### Animal preparation

All experiments were performed with the approval (No. 15032) of the Institutional Animal Care and Use Committee of Showa University, which operates under Japanese Governmental Law (No. 105) for the care and use of laboratory animals. All efforts were made to minimize the suffering and number of animals used. Twenty-one Wistar rats of either sex aged between 21 and 35 days were used in this study. The weights ranged from 80 to 140 g. Preparations were prepared using a method similar to that described by Paton and St. John [18]. The rats were deeply anesthetized with isoflurane until their nociceptive reflexes were abolished. The rats were transected caudal to the diaphragm and submerged in cooled Ringer’s solution (in mM: 125 NaCl, 3 KCl, 24 NaHCO_3_, 1.25 KH_2_PO_4_, 1.25 MgSO_4_, 2.5 CaCl_2_, 10 dextrose). In the cooled Ringer’s solution, the rats were decerebrated at the precollicular level. Preparations were then transferred to a recording chamber, and the descending aorta was cannulated with a double lumen catheter (DLR-4, Braintree Scientific, Braintree, MA). Ringer’s solution containing 1.25% Ficoll (Sigma, St. Louis, MO) and heparin (10 unit/mL) was retrogradely perfused using a roller pump (502S, Watson-Marlow, Falmouth, Cornwall, UK). The perfusate was continuously gassed with 5% CO_2_ and 95% O_2_ and warmed to 31–32°C (temperature measured at the point of entry into the aorta) using an in-line heater (TC324C; Warner Instruments, Hamden, CT). The second lumen of the catheter was used to monitor aortic perfusion pressure. The perfusion pressure was maintained in the range of 35–50 mmHg by adjusting the flow between 25 and 38 mL/min.

### Nerve recording

Preparations were paralyzed using vecuronium bromide (1.5–2 μg/mL; Sigma) prior to the isolation of the peripheral nerves. Using 5 preparations, simultaneous recordings of the motor activity in the RLN, SLN, CN, and PN were obtained with bipolar suction electrodes mounted on separate 3-D micromanipulators. Each peripheral nerve on the left side was isolated, cut distally, and the proximal end was held in the suction electrodes. The PN was isolated from the pleura and cut at the distal end. The CN was identified between the left common carotid artery and sternothyroid muscle. The CN was cut proximally, 1 mm from the left common carotid artery. The SLN and RLN were identified between the trachea and the left common carotid artery. The SLN was cut in the vicinity of the trachea and the RLN was cut at the level of the thyroid gland. Using the other 5 preparations, simultaneous recordings of motor activity in the HGN, SLN, CN, and PN were obtained. The HGN was identified under the mylohyoid muscle and cut immediately before the genioglossus muscle. Using the remaining 5 and 6 preparations, motor activity in the intercostal nerve (ICN) and abdominal nerve (AbN) was recorded with CN and PN motor activity, respectively. The ICN was dissected from between the innermost intercostal muscle and internal intercostal muscle on the left at the 5th–7th thoracic spinal level (T5–T7). The AbN activity was recorded from the 1st lumbar spinal nerve (L1). All signals were amplified using a differential amplifier (DP-304; Warner Instruments), band-pass filtered (1–3k Hz), and stored on a computer using an analog-to-digital converter (CED micro 1401; Cambridge Electronic Design, Cambridge, UK) with version 8 of the Spike2 software (Cambridge Electronic Design). During post-hoc analysis, additional digital filtering was applied using DC remove with a time constant of 0.1 s (Spike2 software) when necessary to remove movement artifact. The nerve activity was rectified and integrated with a time constant of 0.1 s in the Spike2 software.

### Experimental protocol

For hypercapnic stimulation, the perfusate gas composition was altered from normal carbogen (5% CO_2_ and 95% O_2_) to a hypercapnic mixture (8% CO_2_ and 92% O_2_) for 15 min. The pH in the perfusate was altered from 7.4 to 7.2 via changes in the perfusate gas. The perfusate for hypercapnia was prepared in a separate reservoir and was gassed for at least 15 min prior to its administration.

### Data analysis

The following respiratory variables were quantified using the original PN discharges: respiratory frequency, inspiratory duration, and expiratory duration. The duration of phrenic nerve activity above baseline noise was measured as the inspiratory duration per cycle (determined from raw activity). The expiratory duration was the duration of phrenic nerve activity not above the baseline noise per cycle. The integrated nerve activity in each nerve was used to obtain the peak amplitude of the discharge, onset of the discharge, and increasing duration from onset until peak PN discharge (time to peak). These values were obtained using nerve discharges during the last 1 min prior to the changes in the perfusate gas (control), 14–15 min after the perfusate changes (hypercapnia), and 14–15 min after the restoration of the perfusate (recovery). The duration of the pre-inspiratory (pre-I) discharge in the CN and ICN was measured for 5 min to calculate the correlation coefficient. In the experiments in which the AbN activity was recorded, we measured the duration of the CN pre-I discharge, AbN pre-I discharge, and respiratory cycle from 5 min before the perfusate changed to 5 min after the restoration of the perfusate. The respiratory cycle duration was defined as the time from the beginning of one phrenic nerve burst to the next.

Values are presented as the mean ± standard error of the mean (SEM). Data obtained before and after changes in the perfusate gas were subjected to the Wilcoxon signed-rank test or Friedman analysis of variance (ANOVA) test. The Friedman ANOVA was followed by the Wilcoxon singed-rank test when appropriate. Differences in data between groups were analyzed using a two-way ANOVA or Kruskal-Wallis one-way ANOVA. The ANOVA was followed by a Bonferroni post-hoc multiple comparison test when appropriate. Probability values of less than 0.05 were considered significant. Statistical analyses were conducted using SPSS 17.0J (SPSS Japan Inc., Tokyo, Japan) and Microsoft Excel 2011.

## Results

### Respiratory motor activity in the efferent nerves innervating the upper airway muscles in arterially perfused decerebrate rats

**Fig 1:**
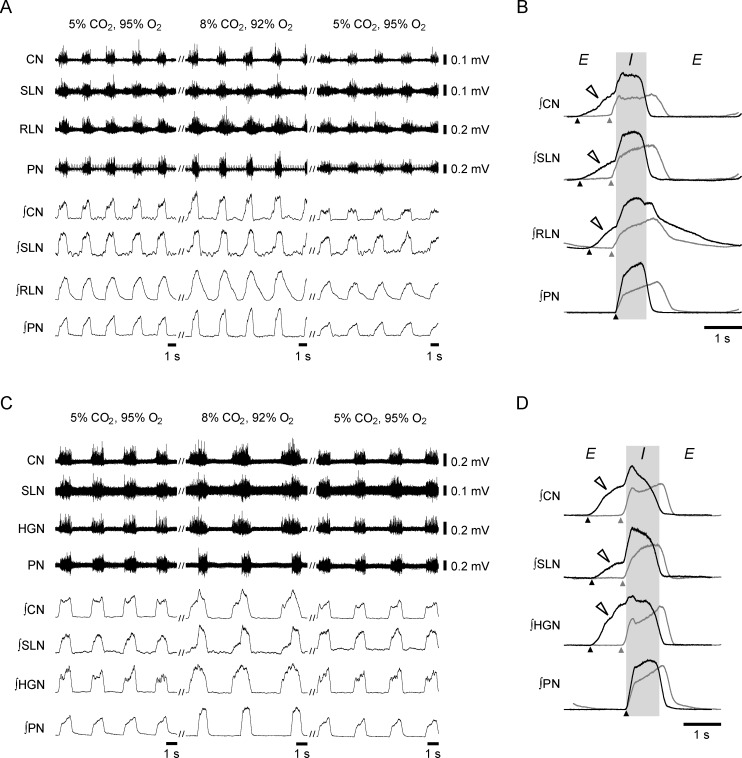
Respiratory motor activity under normocapnic conditions and hypercapnic acidosis. (A) Original and integrated traces of CN, SLN, RLN, and PN activity during normocapnia (5% CO_2_, 95% O_2_), hypercapnia (8% CO_2_, 92% O_2_), and recovery (5% CO_2_, 95% O_2_). (B) Traces averaged from consecutive integrated sweeps for 1 min in the preparation shown in (A). The black and gray traces show the averaged traces during hypercapnia and normocapnia, respectively. The filled arrowheads show the onset of the nerve discharges. The open arrowheads show the pre-inspiration (pre-I) discharges. ‘*I*’ and ‘*E*’ demonstrate the inspiratory and expiratory phases, respectively. (C) Original and integrated traces of the CN, SLN, HGN, and PN activity obtained from a different preparation from (A). (D) Traces averaged from consecutive integrated sweeps for 1 min in the preparation shown in (C).

We first examined the patterns of respiratory motor activity in efferent nerves innervating the upper airway muscles under normocapnic conditions (5% CO_2_) to determine the activation patterns of the upper airway during respiration. In 5 preparations, the motor activities were simultaneously recorded from the CN, SLN, RLN, and PN. The integrated PN activity showed an augmenting inspiratory pattern under normocapnic conditions (Fig 1A), as was previously reported [19]. Discharges in the RLN, SLN, and CN emerged with the PN discharge (Fig 1A); however, their onsets slightly preceded the onset of the PN discharge (Fig 1B, Table 1). The SLN and CN discharges terminated in the inspiration-to-expiration transition; however, the inspiratory activity in the RLN was followed by a decrementing post-inspiratory (post-I) discharge that terminated in the middle of expiration. We next examined the pattern of respiratory motor activity of the HGN, which also innervates the upper airway muscles, in relation to the activity in the SLN, CN, and PN using the other 5 preparations because HGN motor activity has frequently been examined *in situ* in juvenile or adult rats in hypercapnic conditions [9, 20, 21]. The HGN discharge emerged with the PN discharge (Fig 1C). The onset of the HGN discharge also preceded the PN discharge (Fig 1D, Table 1). These results suggest that the upper airway dilates ahead of the contraction of the diaphragm for smooth inspiration.

**Table 1:** The onset of the CN, SLN, HGN and RLN discharges in relation to that of the PN discharge during normocapnia (5% CO_2_) and hypercapnia (8% CO_2_).

		Onset of the pre-I discharge (s)	
	*n*	5% CO_2_, 95% O_2_	8% CO_2_, 92% O_2_
CN	10	-0.26 ± 0.03	-0.85 ± 0.04***
HGN	5	-0.23 ± 0.03	-0.79 ± 0.10*
SLN	10	-0.18 ± 0.02^†^	-0.81 ± 0.03***
RLN	5	-0.14 ± 0.02^††^	-0.42 ± 0.06^†††^^,^ *

The results are presented as the mean ± SE; *n* is the number of preparations examined.

^†^*P* < 0.05

^††^*P* < 0.01

^†††^*P* < 0.001 compared with CN

*P < 0.05 compared with 5% CO_2_

***P < 0.001 compared with 5% CO_2_, two-way ANOVA followed by a Bonferroni post-hoc multiple comparison test.

**Fig 2:**
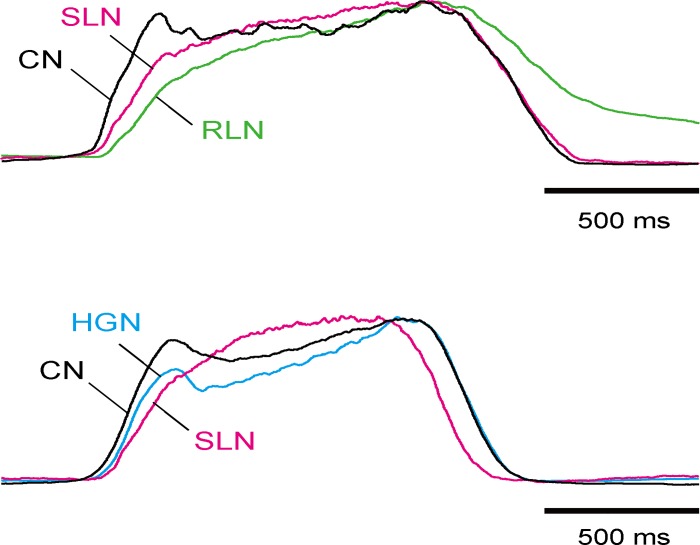
The discharge patterns of the CN, SLN, RLN, and HGN under normocapnic conditions. Averaged traces from two preparations shown in Fig 1 were normalized via the peak amplitude.

We next assessed the discharge patterns in the CN, SLN, RLN, and HGN under normocapnic conditions. The CN discharge started before the other 3 nerves (Fig 2). The HGN discharge started immediately after the CN activity. The time preceding the PN discharge in the HGN discharge did not significantly differ from that in the CN discharge (*P* = 1.000, ANOVA, Fig 2, Table 1). When the traces of the integrated efferent nerve discharges were normalized to their peak amplitudes, the shape of the integrated CN discharge resembled that of the integrated HGN discharge (Fig 2). The activity of the CN and HGN rose more rapidly than that of the RLN and SLN at the beginning of the inspiratory phase, and both displayed two peaks during the inspiratory phase. The SLN and RLN discharges started to emerge after the CN and HGN discharges (Fig 2), and the onset of SLN and RLN activity was significantly later than that of CN activity (SLN: *P* = 0.042, RLN: *P* = 0.003, ANOVA, Table 1). These results suggest that the tongue moves biphasically during inspiration through the coordinated movement of the tongue and infrahyoid muscles, and dilation of the glottis starts after contraction of the lingual radix and fixation of the hyoid bone.

### Respiratory motor activity in the efferent nerves innervating the upper airway muscles during hypercapnic acidosis

When the CO_2_ concentration in the perfusate was increased from 5% to 8% to induce hypercapnic acidosis, the peak amplitude of the PN discharge was significantly increased under hypercapnic conditions (*P* < 0.001, Wilcoxon, Fig 1A, Table 2), as has been previously reported [19]. The respiratory frequency was significantly decreased under hypercapnic conditions (*P* = 0.002, Wilcoxon, Table 2) due to the increase in expiratory duration (*P* < 0.001, Wilcoxon, Table 2). In contrast, the inspiratory duration and the time to peak were shortened under hypercapnic conditions (*P* < 0.001, Wilcoxon, Table 2).

**Table 2:** Comparison of variables of PN activity during normocapnia (5% CO_2_) and hypercapnia (8% CO_2_) (*n* = 21).

	5% CO_2_, 95% O_2_	8% CO_2_, 92% O_2_
Respiratory frequency (Hz)	0.32 ± 0.02	0.27 ± 0.06**
Inspiratory duration (s)	1.06 ± 0.03	0.77 ± 0.03***
Expiratory duration (s)	2.39 ± 0.15	3.05 ± 0.13***
Time to peak (s)	0.84 ± 0.02	0.37 ± 0.03***
Normalized peak amplitude	1.0	1.29 ± 0.06***

The results are presented as the mean ± SE.

**P < 0.01

***P < 0.001 compared with 5% CO_2_, Wilcoxon signed-rank test.

**Fig 3:**
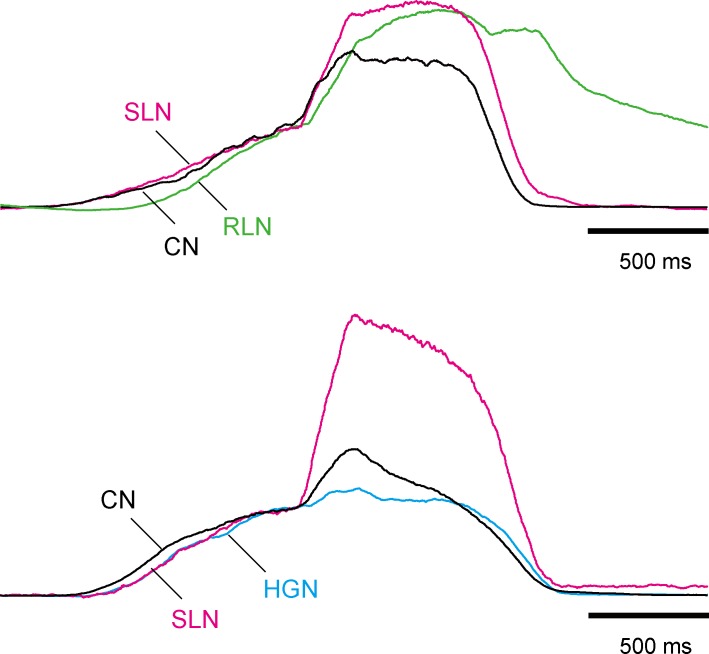
The discharge patterns of the CN, SLN, RLN, and HGN in hypercapnic acidosis. The averaged traces from two preparations shown in Fig 1 were normalized via the peak amplitude of the pre-I discharges.

Hypercapnic acidosis also increased the peak amplitude of the motor activity in all the recorded nerves innervating the upper airway muscles in the inspiratory phase (CN: 141 ± 12.3% of control, *n* = 10, *P* = 0.005; SLN: 137 ± 8.7% of control, *n* = 10, *P* = 0.005; HGN: 127 ± 17.4% of control, *n* = 5, *P* = 0.043; RLN: 161 ± 17.1% of control, *n* = 5, *P* = 0.043, Wilcoxon, Fig 1). There were no significant differences between the degree of increase in each nerve (*P* = 0.227, Kruskal-Wallis). Moreover, the pre-I discharges were induced prior to the PN discharges in all preparations under hypercapnic conditions (Fig 1B and 1D). The duration of the pre-I discharges in all recorded nerves during hypercapnic acidosis was significantly longer than that under normocapnic conditions (CN: *P* < 0.001, SLN: *P* < 0.001, HGN: *P* = 0.026, RLN: *P* = 0.039, ANOVA, Table 1). The pre-I discharges during hypercapnic acidosis were incremental in all these nerves. The HGN and SLN pre-I discharges began immediately after the CN pre-I discharge (Fig 3). The duration of the HGN and SLN pre-I discharges did not significantly differ from that of the CN pre-I discharge (HGN: *P* = 1.000, SLN: *P* = 1.000, ANOVA, Fig 3, Table 1). In contrast, the onset of the RLN discharge significantly lagged behind that of the CN discharge (*P* < 0.001, ANOVA, Fig 3, Table 1). These results suggest that the activity of the upper airway muscles in hypercapnic acidosis differs from that under normocapnic conditions.

### Relationship between the efferent nerve innervating the upper airway muscles and intercostal and abdominal nerves

**Fig 4:**
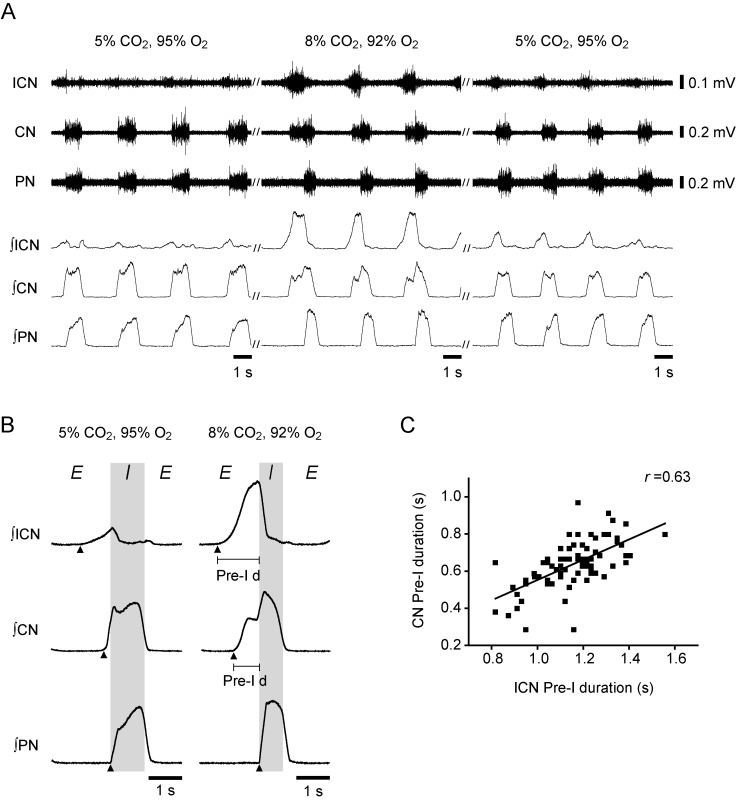
Comparison between intercostal nerve (ICN) activity and CN activity. (A) Original and integrated traces of the ICN, CN, and PN activity during normocapnia (5% CO_2_, 95% O_2_), hypercapnia (8% CO_2_, 92% O_2_), and recovery (5% CO_2_, 95% O_2_). (B) Traces averaged from consecutive integrated sweeps for 1 min in the preparation shown in (A). ‘Pre-I d’ shows the duration of pre-I discharge in the ICN and CN. The filled arrowheads show the onset of the nerve discharges. (C) Correlation between the duration of the CN discharge in the pre-I phase and duration of the ICN discharge in the pre-I phase in the preparation shown in (A).

**Fig 5:**
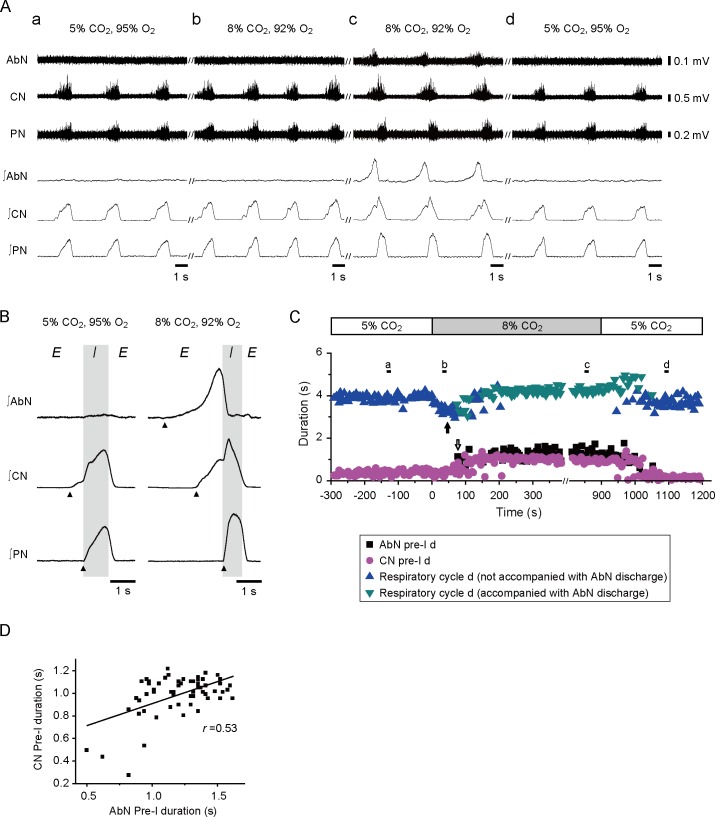
Correlation between abdominal nerve (AbN) activity and CN activity. (A) Original and integrated traces of AbN, CN, and PN activity during normocapnia (5% CO_2_, 95% O_2_), hypercapnia (8% CO_2_, 92% O_2_), and recovery (5% CO_2_, 95% O_2_). (B) Traces averaged from consecutive integrated sweeps for 1 min in the preparation shown in (A). The filled arrowheads show the onset of nerve discharges. (C) Comparison of the duration of the AbN pre-I discharge, CN pre-I discharge, and respiratory cycle in the preparation shown in (A). Black bars show the times that the traces in (A) were recorded. The black arrow shows the time point at which the respiratory duration became shortest. The white arrow shows the time point at which the abdominal discharge appeared. (D) Correlation between the duration of the CN pre-I phase discharge and duration of the AbN pre-I discharge in the preparation shown in (A).

We next examined the relationship between the motor activity of the efferent nerve innervating the upper airway muscles and the motor activity of the ICN innervating the external and internal intercostal muscles, which are typical respiratory muscles. We recorded the nerve activity of the ICN at the level of the 5th or 7th intercostal space simultaneously with the activity of the CN and PN in 5 preparations. Under normocapnic conditions, the ICN exhibited an incrementing low-amplitude pre-I discharge and also exhibited very weak discharge in the post-I phase. Switching to the hypercapnic condition greatly enhanced the pre-I discharge in the ICN in all 5 preparations (Fig 4A and 4B). The duration of the ICN pre-I discharge was positively correlated with the duration of the CN pre-I discharge in hypercapnic acidosis (*r* = 0.63, Fig 4C); however, there was no correlation under normocapnic conditions (*r* = -0.31). Furthermore, we recorded the nerve activity of the AbN at the 1st lumbar level in 6 preparations. Under normocapnic conditions, a distinct discharge was not observed in the AbN (Fig 5A and 5B). In contrast, hypercapnic conditions induced pre-I discharge in the AbN in all 6 preparations (Fig 5A and 5B). Changing to hypercapnic conditions tended to initially decrease the respiratory duration. The respiratory duration became shortest at 50 ± 8.5 s after switching of the perfusate (*n* = 6; Fig 5C). As the AbN pre-I discharge appeared and grew from 102 ± 9.6 s after the switch (*n* = 6), the duration of the CN pre-I discharge and respiratory cycle was prolonged (Fig 5Ac and 5C). The Friedman test revealed that the respiratory duration was significantly different in the last 1 min prior to, 0.5–1.5 min after, and 14–15 min after changing of the perfusate (*P* = 0.030); the difference between the respiratory duration during the last 1 min prior to changing of the perfusate and the duration during the first 0.5–1.5 min after switching approached significance (*P* = 0.056, Wilcoxon). The duration of the CN pre-I discharge and respiratory cycle during the 14–15 min after changing of the perfusate was significantly longer than that during the first 0.5–1.5 min after changing of the perfusate (CN pre-I: *P* = 0.022, respiratory duration: *P* = 0.028, Wilcoxon). The duration of the AbN pre-I discharge was positively correlated with the duration of the CN pre-I discharge in hypercapnic acidosis during the 5 min after changing of the perfusate (*r* = 0.53; Fig 5D). These results suggest that the pre-I activity in the CN in hypercapnic acidosis is due to activation of the expiratory drive.

## Discussion

In the present study, *in situ* arterially perfused decerebrate rat preparations enabled us to study the respiratory activity in the nerves innervating the upper airway muscles without the depressant effects of anesthesia. Some reports have demonstrated that the geniohyoid muscles, which are innervated by the HGN, had no phasic respiratory electromyographic activity in anesthetized cats [22] and young guinea pigs [23]. In contrast, other investigators have demonstrated that the geniohyoid and genioglossus muscles, which are also innervated by the HGN, had phasic inspiratory activity during resting breathing in anesthetized rats [24], rabbits [25], and dogs [26]. It is possible that these inconsistent results might be caused by the differential effects of anesthesia. Since the phasic inspiratory activity pattern in the HGN emerged with the PN discharge in the present study, which was similar to the geniohyoid muscle activity obtained in awake dogs [27] and humans [28], our *in situ* arterially perfused decerebrate rat preparation can be regarded as an appropriate model to study the respiratory function of the upper airway muscles.

The mean duration of PN discharges in our preparations was slightly longer than the discharge duration of the PN that has been suggested as a value (≤1 s) in optimally perfused preparations by Levitt et al. [29] and Bautista and Dutschmann [30]. Furthermore, the post-I discharge in the RLN was relatively weak in our preparation compared with the post-I discharges in the vagus nerve in those studies. Such weak post-I discharge in the RLN was similar to that in the anesthetized *in vivo* rat [31]. Since the integrated phrenic nerve discharge showed a ramping envelope in all recorded preparations and the tissue PO_2_ in the pons in our preparations was 180–250 mmHg (data not shown), which is similar to the PO_2_ level reported by Wilson et al. [32], our preparations were not considered to be in a bad state. However, it is possible that the pontine respiratory groups may not be properly engaged in respiratory rhythm and pattern generation in our preparation because reduction of the excitability in the Kölliker-Fuse nucleus (KF) by activation of μ-opioid receptors in the KF increases inspiratory time owing to loss of post-I drive [29].

In the present study, hypercapnia increased the amplitude of PN activity and decreased inspiratory duration, consistent with the findings in many previous studies *in vivo* [10, 33] and *in situ* [9, 19, 34, 35]. The responses of this preparation to hypercapnia imply an enhancement of inspiration; this notion is reinforced by the decrease in the time to peak PN discharge under hypercapnic acidosis. In contrast, the influence of hypercapnia on the respiratory frequency has differed between studies. In *in vivo* experiments using plethysmography, Holley et al. [36] reported that hypercapnic exposure increased the respiratory frequency in the unanesthetized rat, whereas Wakai et al. [37] reported that hypercapnia does not affect the respiratory frequency in the anesthetized rat. Moreover, Harris and Milsom [13] reported that hypercapnia decreases respiratory frequency during wakefulness but increases it under anesthesia. However, this respiratory reflex to hypercapnia was inhibited after vagotomy. Using artificially perfused *in situ* rat preparations, many previous studies have demonstrated that hypercapnia increases the respiratory frequency [9, 19, 34, 38], whereas some reports indicate that hypercapnia does not significantly affect respiratory frequency [35]. In the present study, after changing the CO_2_ concentration in the perfusate from 5% to 8%, the respiratory frequency initially tended to increase; however, the frequency gradually decreased as the AbN and CN pre-I discharge appeared and grew. Similar relationships between the occurrence of the AbN pre-I discharge and slowing of respiratory rhythm have been reported by Abdala et al. [9] and Molkov et al. [39]. Thus, multiple factors, such as anesthesia, vagotomy, oxygen tension, temperature, types of preparation, etc., probably affect the respiratory response to hypercapnia.

The present study showed that hypercapnic acidosis evoked pre-I discharges in the CN, SLN, and RLN, in addition to the HGN, which has often been shown to display pre-I discharge during hypercapnia [9, 20, 39]. The peak amplitudes of the discharges in these four nerves were also increased. Many studies showed that hypercapnia also evokes a pre-I discharge in the AbN [9, 10, 39, 40]. Pre-I AbN activity functionally constitutes forced expiration, which occurs only under certain conditions such as asphyxia and moderate/intense exercise [9]. In the present study, the ICN and AbN expiratory activity in the pre-I phase was enhanced in a similar manner. Thus, the expiratory drive in the pre-I phase also appears to be augmented by hypercapnic acidosis in the present study. Moreover, the duration of the CN pre-I discharge was positively correlated with the duration of the ICN pre-I discharge. These observations raise the possibility that the expiratory drive from the respiratory network enhanced by hypercapnia may be the source of the pre-I discharges in the CN, HGN, SLN, and RLN during forced expiration.

The RLN includes both efferents innervating the laryngeal abductors and laryngeal adductors. Dutschmann et al. [41] assumed that RLN activity in the inspiratory phase and the post-I phase likely represented the activity of the abductors and adductors, respectively, because the subglottal pressure decreases during the inspiratory phase and abruptly increases with the start of the post-I phase. The abductor of the glottis has been reported to contract simultaneously with the abdominal muscles during forced expiration, such as when coughing [42, 43]. Therefore, the RLN activity in the pre-I phase during the forced expiration evoked by hypercapnia most likely reflects the activity of the abductor of the glottis. Because the CN innervates the infrahyoid muscles, the enhanced activity of the CN during the pre-I phase is likely to facilitate jaw opening by fixing the hyoid bone, leading to the securing of the airway. Such securing of the airway that occurs at a certain time before the dilation of the glottis may facilitate ventilation to improve hypercapnic acidosis. Furthermore, in the present study, hypercapnia enhanced the peak amplitudes of the discharges during the inspiratory phase in these 4 nerves in addition to recruitment of the pre-I discharge. Thus, under hypercapnia, the airway resistance was reduced during both pre-I and the succeeding inspiratory phases in our preparation, as indicated by Abdala et al. [9]. Such activity of the upper airway muscles innervated by the CN, SLN, RLN, and HGN can facilitate air ventilation during the pre-I and the succeeding inspiratory phases. Activation of the alae nasi, dilator muscles of the upper airway located in the nares, has been reported to precede the onset of inspiratory airflow and be increased in the inspiratory phase during CO_2_-induced hyperpnea in normal humans [44] and during sleep apnea in patients with obstructive sleep apnea [45]. Such a phenomenon is similar to the findings of the present study. The basic knowledge regarding the efferent nerve activity of the upper airway obtained in this study may be useful in elucidating the mechanisms of upper airway obstruction under general anesthesia or sleep-related breathing disorders.
